# Cadmium Exposure and Hypertension in the 1999–2004 National Health and Nutrition Examination Survey (NHANES)

**DOI:** 10.1289/ehp.10764

**Published:** 2007-10-30

**Authors:** Maria Tellez-Plaza, Ana Navas-Acien, Ciprian M. Crainiceanu, Eliseo Guallar

**Affiliations:** 1 Department of Environmental Health Sciences and; 2 Department of Epidemiology, Johns Hopkins University Bloomberg School of Public Health, Baltimore, Maryland, USA; 3 Department of Cardiovascular Epidemiology and Population Genetics, Centro Nacional de Investigaciones Cardiovasculares (CNIC), Madrid, Spain; 4 Department of Biostatistics, Johns Hopkins University Bloomberg School of Public Health, Baltimore, Maryland, USA; 5 Departments of Medicine and Welch Center for Prevention, Epidemiology and Clinical Research, Johns Hopkins Medical Institutions, Baltimore, Maryland, USA

**Keywords:** Blood pressure, cadmium, hypertension, NHANES, smoking

## Abstract

**Introduction:**

Cadmium induces hypertension in animal models. Epidemiologic studies of cadmium exposure and hypertension, however, have been inconsistent.

**Objective:**

We aimed to investigate the association of blood and urine cadmium with blood pressure levels and with the prevalence of hypertension in U.S. adults who participated in the 1999–2004 National Health and Nutrition Examination Survey (NHANES).

**Methods:**

We studied participants ≥ 20 years of age with determinations of cadmium in blood (*n* = 10,991) and urine (*n* = 3,496). Blood and urine cadmium were measured by atomic absorption spectrometry and inductively coupled plasma–mass spectrometry, respectively. Systolic and diastolic blood pressure levels were measured using a standardized protocol.

**Results:**

The geometric means of blood and urine cadmium were 3.77 nmol/L and 2.46 nmol/L, respectively. After multivariable adjustment, the average differences in systolic and diastolic blood pressure comparing participants in the 90th vs. 10th percentile of the blood cadmium distribution were 1.36 mmHg [95% confidence interval (CI), −0.28 to 3.00] and 1.68 mmHg (95% CI, 0.57–2.78), respectively. The corresponding differences were 2.35 mmHg and 3.27 mmHg among never smokers, 1.69 mmHg and 1.55 mmHg among former smokers, and 0.02 mmHg and 0.69 mmHg among current smokers. No association was observed for urine cadmium with blood pressure levels, or for blood and urine cadmium with the prevalence of hypertension.

**Conclusions:**

Cadmium levels in blood, but not in urine, were associated with a modest elevation in blood pressure levels. The association was stronger among never smokers, intermediate among former smokers, and small or null among current smokers. Our findings add to the concern of renal and cardiovascular cadmium toxicity at chronic low levels of exposure in the general population.

Cadmium is a toxic and carcinogenic metal widely distributed in the environment [Agency for Toxic Substances and Disease Registry (ATSDR) 1999; International Agency for Research on Cancer (IARC) 1993; Nordberg et al. 2007). In the general population, the primary sources of cadmium exposure are cigarette smoke, food intake (shellfish, offal, certain vegetables), and ambient air particularly in urban areas and in the vicinity of industrial settings. Cadmium exposure induces hypertension in animal models (Satarug et al. 2006; Schroeder and Vinton 1962). In occupationally and environmentally exposed populations, cadmium is nephrotoxic, inducing tubular and glomerular dysfunction (Åkesson et al. 2005; ATSDR 1999; Jin et al. 2004). Epidemiologic studies of the association of environmental cadmium exposure with blood pressure end points are inconsistent. Although some studies found positive associations (McKenzie and Kay 1973; Pizent et al. 2001; Vivoli et al. 1989; Whittemore et al. 1991), other studies found null or even inverse associations (Beevers et al. 1976; Kagamimori et al. 1986; Staessen et al. 1984, 1991). Some epidemiologic studies have several strengths including prospective designs (Staessen et al. 2000); many studies, however, have been limited by small sample sizes, lack of adjustment for potential confounders, lack of standardization of blood pressure measurements, or other methodologic limitations.

The objective of our study was to investigate the association of blood and urine cadmium levels with blood pressure levels and with the prevalence of hypertension in a representative sample of U.S. adults who participated in the 1999–2004 National Health and Nutrition Examination Survey (NHANES). Urine and blood cadmium are biomarkers of ongoing and long-term cadmium exposure. The biological half-life of cadmium is very long (15–30 years), and it progressively accumulates in the kidneys and other organs (ATSDR 1999; Nordberg et al. 2007). Urine cadmium is considered the biomarker of choice to assess chronic exposure because cadmium in urine more readily reflects cadmium concentrations in the renal cortex (ATSDR 1999; Nordberg et al. 2007). Blood cadmium also reflects long-term exposure, but it is more influenced by recent exposure (ATSDR 1999; Elinder et al. 1988; Jarup et al. 1983; Nordberg et al. 2007).

## Methods

### Study population

NHANES 1999–2004, conducted by the U.S. National Center for Health Statistics [NCHS; Centers for Disease Control and Prevention (CDC), Atlanta, GA], used a complex multistage sampling design to obtain a representative sample of the civilian noninstitutionalized U.S. population. For the present analysis we used data from 15,332 adults ≥ 20 years of age who participated in the NHANES 1999–2004 interviews and physical examinations. The overall participation rate was 70.3% (NCHS 2007a). We further excluded 833 pregnant women, 1,719 participants missing blood cadmium, 582 participants missing blood pressure measurements, 100 participants with diastolic blood pressure levels equal to zero, and 1,107 participants missing other variables of interest, leaving 10,991 eligible participants for analyses based on blood cadmium. NHANES 1999–2004 measured urine metals in a third random sample of the study population, leaving 3,496 participants available for analyses based on urine cadmium. NHANES 1999–2004 interviews, physical examinations, and written informed consents were approved by the NCHS Institutional Review Board.

### Blood and urine cadmium

Blood and urine cadmium were measured at the Environmental Health Sciences Laboratory of the CDC National Center for Environmental Health (NCEH) after confirmation of no background contamination in all collection and storage materials and using extensive quality control procedures (NCHS 2006a, 2007b, 2007c).

Cadmium levels in whole blood were measured on a Perkin-Elmer model SIMAA 6000 (PerkinElmer, Norwalk, CT) simultaneous multielement atomic absorption spectrometer, with Zeeman background correction (NCHS 2004a, 2005a, 2006b). The limit of detection was 2.67 nmol/L in NHANES 1999–2002 and 1.78 in NHANES 2003–2004. Of the study participants, 21.6% and 13.4% had blood cadmium levels below the limits of detection in NHANES 1999–2002 and NHANES 2003–2004, respectively. National Institute of Standards and Technology (NIST) whole-blood standard reference materials were used for external calibration (NCHS 2006a, 2007b, 2007c). The interassay coefficients of variation ranged from 4.1% to 9.4%.

Cadmium levels in spot urine specimens were measured by inductively coupled plasma-mass spectrometry (PerkinElmer/SCIEX model 500; PerkinElmer, Shelton, CT) using a multielement analytical technique (NCHS 2004a, 2005a, 2006b). In NHANES 1999–2002, cadmium levels in urine were corrected for interference from molybdenum oxide. A total of 3.3% of participants had urine cadmium levels below the limit of detection (0.53 nmol/L). NIST urine standard reference material 2679 was used for external calibration, and spiked pools prepared at the laboratory were used for internal quality control. Quality control samples included both bench and blind samples (NCHS 2006a, 2007b, 2007c). The inter-assay coefficients of variation ranged from 1.2% to 4.7%. For blood and urine cadmium levels below the limit of detection, NHANES reported a level equal to the limit of detection divided by the square root of 2.

### Blood pressure

A specific protocol was used to measure blood pressure in NHANES 1999–2004. Two physicians and two health technologists were trained to measure blood pressure using a standardized protocol that followed the American Heart Association guidelines (NCHS 2002a, 2004b, 2005b). Three and sometimes four systolic and diastolic blood pressure determinations were taken on the same day in a sitting position. Blood pressure determinations were taken after 5 min rest using a mercury sphygmomanometer, with an appropriate size cuff (five sizes available) placed on the bared right arm.

Quality control and assurance procedures included extensive initial training, quarterly recertification, procedural checklists, and continuous review of data for systematic error (Ostchega et al. 2003). Mean systolic and diastolic blood pressure were computed discarding the first reading, except when only one reading was available (NCHS 2002a, 2004b, 2005b). Hypertension was defined as a mean systolic blood pressure ≥ 140 mmHg, a mean diastolic blood pressure ≥ 90 mmHg, a self-reported physician diagnosis, or medication use.

### Other variables

**Figure 1:**
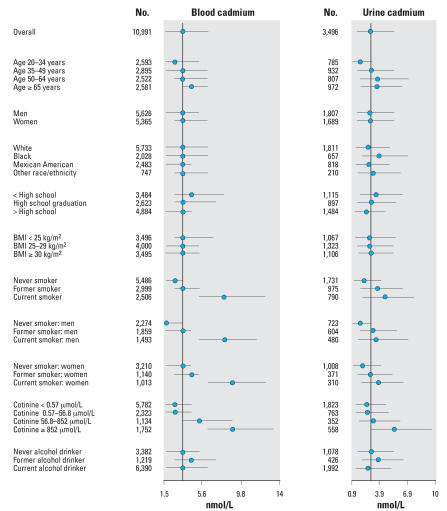
Blood and urine cadmium median (interquartile range) levels by participant characteristics. Points represent medians; horizontal lines represent interquartile ranges; and the dotted vertical line represents the median for the overall study sample.

Information on age, sex, race/ethnicity, education, smoking, and alcohol consumption was based on self-report (Table 1, Figure 1). Body mass index (BMI) was calculated by dividing measured weight in kilograms by measured height in meters squared. Serum cotinine was measured by an isotope-dilution high-performance liquid chromatography/atmospheric pressure chemical ionization tandem mass spectrometric method (NCHS 2006a, 2007b, 2007c). Serum creatinine was measured by the modified kinetic method of Jaffé and corrected for NHANES 1999–2000 as recommended in the study protocol (NCHS 2006a, 2007b, 2007c). Estimated glomerular filtration rate was calculated from calibrated creatinine, age, sex, and race/ethnicity using the Modification of Diet in Renal Disease Study formula (Stevens et al. 2006). Blood lead levels were measured simultaneously to blood cadmium levels using multielement atomic absorption spectrometry with Zeeman background correction (NCHS 2006a, 2007b, 2007c). Urine creatinine was determined using a Jaffé rate reaction measured with a CX3 analyzer (NCHS 2006a, 2007b, 2007c).

### Statistical analysis

All statistical analyses were performed using the survey package in the R statistical language to account for the complex sampling design and weights in NHANES 1999–2004 and to obtain appropriate standard errors for all estimates (Lumley 2007). Cadmium levels in blood and urine and lead levels in blood were right skewed and log-transformed for analyses. We estimated adjusted mean differences in blood pressure levels or adjusted odds ratios (ORs) for the prevalence of hypertension comparing quartiles 2 to 4 of cadmium to the lowest quartile using linear and logistic regression, respectively. Quartile cutoffs were based on weighted distributions in the whole study sample. In addition to quartiles, we also used linear and logistic models to compare the 90th with the 10th percentile of cadmium distribution, assuming a log-linear relationship. Statistical models were initially adjusted for age (restricted cubic spline transformation), sex, race/ethnicity and education. We further adjusted for smoking (never, former, current, and serum cotinine), alcohol intake (never, former, current), BMI, use of antihypertensive medication, menopause status, and lead exposure. Adjustment for estimated glomerular filtration rate did not affect the conclusions (data not shown). For urine cadmium, all models were adjusted for urine creatinine to account for variations in dilution in spot urine samples (Barr et al. 2005). *p*-Values for linear trend were obtained by including log-transformed cadmium levels as continuous variables in the regression models.

We also evaluated the association of blood and urine cadmium with blood pressure end points for subgroups defined by sex and smoking status. *p*-Values for the interactions between blood and urine cadmium with participant characteristics were obtained from adding an interaction term between log-transformed cadmium (blood, urine) and the corresponding participant characteristic (sex, smoking status).

## Results

The geometric means of blood and urine cadmium were 3.77 nmol/L (0.42 μg/L) and 2.46 nmol/L (0.3 μg/L), respectively (Table 1). The Pearson correlation coefficient between blood and urine log-cadmium levels was 0. 42 (*p* < 0.001). Blood and urine cadmium levels were higher in older participants, in participants with lower education, and in current and former smokers (Figure 1). By smoking status category (never, current, former), blood and urine cadmium levels were generally higher in women than in men. Blood cadmium levels were more strongly correlated with current smoking and with serum cotinine than were urine cadmium levels. The weighted prevalence of hypertension in the study sample was 35%.

After multivariable adjustment, the average differences in systolic and diastolic blood pressure comparing the highest to the lowest quartiles of blood cadmium were 1.50 mmHg (95% CI, −0.24 to 3.24) and 1.23 mmHg (95% CI, 0.11 to 2.35), respectively (Table 2, model 2). Compared with participants in the 10th percentile of the blood cadmium distribution, participants in the 90th percentile had 1.36 mmHg (95% CI, −0.28 to 3.00) higher systolic blood pressure levels and 1.68 mmHg (95% CI, 0.57 to 2.78) higher diastolic blood pressure levels. No association was observed for urine cadmium.

The multivariable adjusted OR for hypertension comparing the highest to the lowest quartile of blood cadmium was 1.03 (95% CI, 0.77 to 1.36) (Table 3). The corresponding OR comparing participants in the 90th with the 10th percentile of the blood cadmium distribution was 1.14 (95% CI, 0.89 to 1.45). Similarly, no association was observed between urine cadmium and the prevalence of hypertension.

By sex, the associations of blood and urine cadmium with blood pressure end points were markedly similar for men and women, and none of the interactions were statistically significant (Table 4). By smoking status, the average differences in systolic and diastolic blood pressure levels comparing participants in the 90th with the 10th percentile of the blood cadmium distribution were 2.35 mmHg and 3.27 mmHg, respectively, among never smokers; 1.69 mmHg and 1.55 mmHg among former smokers; and 0.02 mmHg and 0.69 mmHg among current smokers. The modification of the associations between blood cadmium levels with systolic and diastolic blood pressure levels were statistically significant for current smokers compared with never smokers. For urine cadmium, the associations by smoking status were similar for all subgroups.

## Discussion

In a representative sample of U.S. adults who participated in NHANES 1999–2004, cadmium levels in blood, but not in urine, were associated with a modest elevation of blood pressure levels. There was no association between cadmium levels and the prevalence of hypertension. By smoking status, the association for blood cadmium and blood pressure levels was stronger among never smokers, intermediate among former smokers, and small or null among current smokers. Both urine and blood cadmium are biomarkers of long-term and ongoing cadmium exposure, although blood cadmium reflects recent exposure better than urine cadmium (ATSDR 1999; Elinder et al. 1988; Jarup et al. 1983). In our study, this was confirmed by a stronger association of blood cadmium compared with urine cadmium with current smoking status and serum cotinine, a biomarker or recent smoking. Our findings of a positive association of blood cadmium, but not urine cadmium, with blood pressure may indicate that blood pressure levels are affected by recent rather than long-term cadmium exposure. Alternatively, it is possible that blood cadmium reflects biologically active cadmium better than urine cadmium.

Few epidemiologic studies have measured both blood and urine cadmium levels. In Belgium, the CadmiBel study assessed the health consequences of environmental cadmium contamination (Staessen et al. 1991, 2000). In a prospective analysis of 336 men and 356 women residing in the two rural areas of the CadmiBel study (Staessen et al. 2000), changes in blood cadmium levels between 1985–1989 (baseline) and 1991–1995 (follow-up) were positively associated with changes in systolic and diastolic blood pressure, although the association was statistically significant only for diastolic blood pressure among women. Similar to our study, no association was found between urine cadmium and blood pressure levels. The relative risk for developing definite hypertension for a doubling of baseline blood cadmium levels was 1.28 (95% CI, 0.87 to 1.88), and for a doubling of baseline urine cadmium levels it was 1.16 (95% CI, 0.84 to 1.62) (Staessen et al. 2000). Because blood or urine cadmium were not associated with increased blood pressure levels in cross-sectional analyses of all CadmiBel study participants (*n =* 2,086) (Staessen et al. 1991) and the prospective association was only statistically significant for diastolic blood pressure among women (Staessen et al. 2000), the prospective association between changes in blood cadmium and blood pressure found in this study were considered uncertain.

Other studies of cadmium and blood pressure have measured only urine or blood cadmium, with inconsistent findings. In the United States, a subsample of 951 adults who participated in NHANES II (1976–1988) found a positive but modest association of urine cadmium with blood pressure levels (Whittemore et al. 1991). Other studies have been smaller and their findings were subject to substantial random variability (Beevers et al. 1976; McKenzie and Kay 1973; Pizent et al. 2001; Vivoli et al. 1989; Whittemore et al. 1991). Finally, in a cadmium-polluted area in Japan, 52 women with *Itai-Itai* disease had lower systolic and diastolic blood pressure levels compared with 104 age-matched women living out of the cadmium-polluted area (Kagamimori et al. 1986). Indeed, despite important nephrotoxicity, hypertension has not been reported as a typical finding in *Itai-Itai* disease patients in the cadmium-polluted area of Japan (Nordberg et al. 2007). The possible relevance of findings from populations heavily exposed to cadmium to explain the null or even possible inverse association between urine cadmium levels and blood pressure end points in our study is unknown.

Cadmium exposure induces hypertension in animal models (Satarug et al. 2006), although the mechanisms for cadmium-related hypertension remain unclear. A primary mechanism for cadmium toxicity is depletion of glutathione and alteration of sulfhydryl homeostasis (Valko et al. 2005), thus indirectly increasing oxidative stress and lipid peroxidation (Yiin et al. 1999). Cadmium induces renal proximal tubular injury, salt retention, and volume overload which may produce hypertension (Satarug et al. 2006). Other potential mechanisms include partial agonism for calcium channels (Varoni et al. 2003), direct vasoconstrictor action, activation of the sympathetic nervous system, and inhibition of vasodilator substances such as nitric oxide (Bilgen et al. 2003; Varoni et al. 2003). Because cadmium levels used in experimental models are much higher than exposure in the U.S. general population, the relevance of these mechanisms to human hypertension is uncertain.

Cadmium is absorbed through the respiratory and digestive tracts. Under conditions of chronic exposure, cadmium is transported in blood bounded mainly to metallothionein. Metallothionein is a low-molecular-weight metal-binding protein induced by cadmium exposure that plays an important role in cadmium metabolism and toxicokinetics (Nordberg et al. 1992, 2007). Induction of metallothionein depends on the dose and frequency of cadmium exposure. For instance, smokers may be more likely to induce metallothionein because they are repeatedly exposed to cadmium from cigarette smoke. By binding cadmium, metallothionein may protect the kidneys and other organs from the toxic effects of cadmium (Nordberg et al. 1992). In the renal cortex, cadmium–metallothionein compounds are stored in tubular cells with only a minor proportion of the body burden being excreted through urine (ATSDR 1999). As a result, cadmium progressively accumulates with age in the kidney and other organs, although autopsy studies have shown that cadmium concentration in the kidneys decreases after 45–50 years of age (Nordberg et al. 2007; Satarug et al. 2003; Travis and Haddock 1980). Although fluctuations in cadmium exposure result in blood cadmium fluctuations, few changes are observed in urine cadmium which, in the absence of tubular damage, reflects cadmium accumulation in the body over time (Nordberg et al. 1992). Experimental studies at low exposure levels are needed to determine the impact of short- versus long-term cadmium exposure on blood pressure and the relevance of metallothionein induction.

The findings of our study must be interpreted with caution. First, single blood and urine cadmium measurements may be limited to fully assess cadmium exposure and cadmium internal dose. Cadmium levels in blood were low, close to the instrument limit of detection and with most participants having levels over a small number of values. For urine cadmium, the use of spot urine samples and the limitations of using creatinine to adjust for urine dilution (Barr et al. 2005) may have amplified this problem. Blood and urine cadmium levels are thus subject to substantial within-person error and may also be affected by other participants’ characteristics. Given that cadmium is a well-established nephrotoxicant even at low levels of chronic exposure (Åkesson et al. 2005), underestimation of the potential effects of cadmium on blood pressure and hypertension is a concern. Metallothionein levels, which could explain part of the variability of the association of cadmium with blood pressure end points, were not determined in NHANES.

Second, additional limitations of our study include the cross-sectional design and the difficulty in adjusting for some potential confounders, including socioeconomic status, education, or other exposures that may occur in the same environmental settings. However, our results persisted after adjustment for educational level, race/ethnicity, smoking status, serum cotinine levels, and blood lead levels.

Finally, the modification of the association of blood cadmium with blood pressure levels by smoking status was the result of a post hoc analysis. Potential explanations include markedly different sources, routes, and patterns of cadmium exposure for smokers versus nonsmokers, unknown differences in participant characteristics or co-exposures by smoking status, and random variability. Among smokers, blood cadmium is likely to be a marker of smoking intensity, and other components in tobacco smoke could contribute to these differences. Indeed, smokers have generally lower blood pressure levels than do nonsmokers (Green et al. 1986; Mikkelsen et al. 1997), and among smokers, higher levels of serum cotinine have been associated with lower blood pressure levels (Benowitz and Sharp 1989).

## Conclusion

At the low levels of cadmium exposure observed in NHANES 1999–2004, we found a modest but positive association between blood cadmium and blood pressure levels. The association of blood cadmium but not of urine cadmium with elevated blood pressure could be related to recent cadmium exposure having a short-term effect on blood pressure levels. Also, blood cadmium could more readily reflect biologically active cadmium. These interpretations require testing in experimental settings at the relevant levels of exposure. Our findings add to the concern on renal and cardiovascular cadmium toxicity at chronic low levels of exposure (Åkesson et al. 2005; ATSDR 1999; Jin et al. 2004; Navas-Acien et al. 2004, 2005; Nordberg et al. 2007), and support the need for efforts to reduce environmental cadmium exposure in the general population.

## Figures and Tables

**Table 1:** Participant characteristics by hypertension status.^a^

Characteristic	Hypertension (*n* = 4,669)	No hypertension (*n* = 6,322)	All (*n* = 10,991)
Age (years)	56.4 (0.36)	40.9 (0.25)	46.5 (0.27)
Sex (% male)	48 (0.93)	51 (0.56)	50.0 (0.45)
Race/ethnicity (% white)	76 (1.8)	73 (1.6)	74 (1.6)
Education (% > high school)	48 (1.1)	58 (1.2)	54 (1.0)
BMI (kg/m^2^)	29.9 (0.15)	26.9 (0.10)	28.0 (0.10)
Smoking			
Former smoker (%)	33 (0.90)	21 (0.84)	25 (0.7)
Current smoker (%)	19 (0.74)	28 (1.0)	25 (0.7)
Cotinine^b^ (nmol/L)	2.23 (1.84–2.70)	4.36 (3.52–5.61)	3.48 (2.87–4.24)
Alcohol intake			
Former drinker (%)	12 (0.97)	7 (0.53)	9 (0.6)
Current drinker (%)	54 (1.8)	69 (1.5)	63 (1.6)
Glomerular filtration rate (1 mL/min/1.73 m^2^)	84.5 (0.53)	95.6 (0.51)	91.7 (0.46)
Blood lead^b^ (μmol/L)	0.088 (0.085–0.091)	0.074 (0.072–0.076)	0.079 (0.077–0.081)
Blood cadmium^b^ (nmol/L)	3.99 (3.85–4.12)	3.66 (3.49–3.83)	3.77 (3.63–3.92)
Urine cadmium^b^,^c^ (nmol/L)	2.75 (2.58–2.93)	2.32 (2.18–2.47)	2.46 (2.35–2.59)
Urine cadmium^b^,^c^ (nmol/mmol creatinine)	0.34 (0.32–0.36)	0.24 (0.23–0.25)	0.27 (0.26–0.28)

To convert serum cotinine from nmol/L to ng/mL, divide by 5.68; blood lead from μmol/L to μg/dL, divide by 0.0483; and blood and urine cadmium from nmol/L to μg/L, divide by 8.897. To convert urine cadmium from nmol/mmol creatinine to μg/g creatinine, divide by 1.006.

a Hypertension defined as mean systolic blood pressure ≥ 140 mmHg, mean diastolic blood pressure ≥ 90 mmHg, self-reported physician diagnosis, or medication use.

b Geometric mean (95% CI); other results in the table are arithmetic means or percentages (SE).

c Subsample (hypertension = 1,515; no hypertension=1,981; all = 3,496).

**Table 2:** Change (95% CI) of systolic and diastolic blood pressure levels by blood cadmium and urine cadmium levels (nmol/L).

		Systolic blood pressure (mmHg)		Diastolic blood pressure (mmHg)	
	No.	Model 1	Model 2	Model 1	Model 2
Blood cadmium					
Quartile 1 (≤ 1.78)	2,508	0.00 (reference)	0.00 (reference)	0.00 (reference)	0.00 (reference)
Quartile 2 (1.78–3.56)	3,394	0.21 (−0.63 to 1.06)	0.72 (−0.11 to 1.57)	0.60 (−0.10 to 1.31)	1.00 (0.28 to 1.71)
Quartile 3 (3.56–6.23)	2,821	0.87 (−0.36 to 2.11)	1.85 (0.52 to 3.19)	0.92 (−0.07 to 1.91)	2.01 (0.86 to 3.15)
Quartile 4 (> 6.23)	2,268	−0.24 (−1.40 to 0.89)	1.50 (−0.24 to 3.24)	−1.06 (−1.80 to −0.32)	1.23 (0.10 to 2.35)
*p-*Trend		0.775	0.116	0.080	0.006
90th–10th percentile		−0.15 (−1.18 to 0.88)	1.36 (−0.28 to 3.00)	−0.63 (−1.31 to 0.05)	1.68 (0.57 to 2.78)
Urine cadmium^a^,^b^					
Quartile 1 (≤ 1.51)	852	0.00 (reference)	0.00 (reference)	0.00 (reference)	0.00 (reference)
Quartile 2 (1.51–2.93)	895	−0.92 (−2.35 to 0.51)	−0.89 (−2.47 to 0.69)	−0.70 (−1.65 to 0.23)	−0.26 (−1.28 to 0.75)
Quartile 3 (2.93–5.51)	881	−1.01 (−3.25 to 1.23)	−0.55 (−3.03 to 1.93)	−0.63 (−1.78 to 0.51)	0.26 (−0.94 to 1.48)
Quartile 4 (> 5.51)	868	−2.90 (−5.32 to −0.48)	−2.05 (−5.11 to 0.99)	−2.01 (−3.68 to −0.33)	−0.45 (−2.34 to 1.44)
*p-*Trend		0.031	0.251	0.005	0.565
90th–10th percentile		−2.92 (−5.47 to −0.37)	−1.78 (−4.76 to 1.19)	−2.10 (−3.49 to −0.73)	−0.44 (−1.94 to 1.05)

Model 1 was adjusted for age (years modeled as restricted cubic spline with 5 knots), sex, race/ethnicity, education (< high school, high school, > high school). Model 2 was further adjusted for smoking status (never, former, current), cotinine (log_10_ nmol/L), alcohol intake (never, former, current), BMI (kg/m^2^), menopause status (yes, no), antihypertensive medication (yes, no), blood lead (log_10_ μmol/L)

a All models for urine cadmium are adjusted for urine creatinine levels.

b Subsample (*n* = 3,496).

**Table 3:** ORs (95% CIs) of hypertension by quartile of cadmium in U.S. adults (nmol/L).

	Cases (*n*)	Noncases (*n*)	Model 1	Model 2
Blood cadmium				
Quartile 1 (≤ 1.78)	819	1,689	1.00 (reference)	1.00 (reference)
Quartile 2 (1.78–3.56)	1,419	1,975	0.89 (0.75 to 1.05)	0.98 (0.80 to 1.19)
Quartile 3 (3.56–6.23)	1,452	1,369	0.99 (0.85 to 1.16)	1.25 (0.98 to 1.59)
Quartile 4 (> 6.23)	979	1,289	0.77 (0.66 to 0.90)	1.03 (0.77 to 1.36)
*p-*Trend			0.005	0.303
90th–10th percentile			0.84 (0.75 to 0.94)	1.14 (0.89 to 1.45)
Urine cadmium^a^,^b^				
Quartile 1 (≤ 1.51)	301	551	1.00 (reference)	1.00 (reference)
Quartile 2 (1.51–2.93)	369	526	0.86 (0.64 to 1.17)	0.80 (0.54 to 1.21)
Quartile 3 (2.93–5.51)	430	451	0.93 (0.65 to 1.36)	1.02 (0.66 to 1.58)
Quartile 4 (> 5.51)	415	453	0.68 (0.48 to 0.97)	0.72 (0.43 to 1.21)
*p-*Trend			0.021	0.170
90th–10th percentile			0.63 (0.43 to 0.91)	0.66 (0.37 to 1.17)

Model 1 was adjusted for age (years modeled as restricted cubic splines with 5 knots), sex, race/ethnicity, education (< high school, high school, > high school). Model 2 was further adjusted for smoking (never, former, current), cotinine (log_10_ nmol/L), alcohol intake (never, former, current), BMI (kg/m^2^), menopause (yes, no), antihypertensive medication (yes, no), blood lead (log_10_ μmol/L).

a All models for urine cadmium are adjusted for urine creatinine levels.

b Subsample (*n* = 3,496).

**Table 4:** Change (95% CI) of systolic and diastolic blood pressure levels (mmHg) and ORs (95% CIs) of hypertension comparing the 90th to 10th percentile of blood and urine cadmium levels by sex and smoking status.

	No.	Systolic blood pressure (mmHg)	*p*-Value for interaction	Diastolic blood pressure (mmHg)	*p*-Value for interaction	OR of hypertension	*p*-Value for interaction
Blood cadmium							
Sex							
Men	5,626	0.86 (−0.84 to 2.56)	Ref.	1.81 (0.40 to 3.22)	Ref.	1.00 (0.74 to 1.37)	Ref.
Women	5,365	1.40 (−0.82 to 3.62)	0.444	1.78 (0.65 to 2.92)	0.495	1.31 (0.90 to 1.90)	0.487
Smoking							
Never	5,486	2.35 (0.64 to 4.05)	Ref.	3.27 (1.69 to 4.84)	Ref.	1.25 (0.87 to 1.81)	Ref.
Former	2,999	1.69 (−1.55 to 4.92)	0.200	1.55 (−0.39 to 3.49)	0.070	1.23 (0.72 to 2.07)	0.491
Current	2,506	0.02 (−1.97 to 2.01)	0.001	0.69 (−0.69 to 2.06)	0.006	1.01 (0.72 to 1.44)	0.245
Urine cadmium^a^,^b^							
Sex							
Men	1,807	−3.27 (−5.48 to −1.05)	Ref.	−1.74 (−3.38 to −0.09)	Ref.	0.60 (0.34 to 1.08)	Ref.
Women	1,689	−4.68 (−6.94 to −2.43)	0.926	−1.00 (−2.39 to 0.39)	0.595	0.51 (0.33 to 0.79)	0.762
Smoking							
Never	1,731	−4.06 (−6.89 to −1.23)	Ref.	−1.08 (−2.69 to 0.53)	Ref.	0.34 (0.19 to 0.62)	Ref.
Former	975	−3.10 (−7.20 to 0.99)	0.996	−0.36 (−3.03 to 2.31)	0.948	0.89 (0.42 to 1.87)	0.123
Current	790	−4.72 (−8.27 to −1.18)	0.730	−2.67 (−5.22 to −0.11)	0.312	0.75 (0.41 to 1.38)	0.100

Models were adjusted for age (years modeled as restricted cubic splines with 5 knots), race/ethnicity, education (< high school, high school, > high school), cotinine (log10 nmol/L), alcohol intake (never, former, current), BMI (kg/m^2^), menopause status (yes, no), antihypertensive medication (yes, no, only for systolic and diastolic blood pressure models), blood lead (log_10_ μmol/L), sex (models by smoking status), or smoking status (models by sex).

a All models for urine cadmium are adjusted for urine creatinine levels.

b Subsample (*n* = 3,496).
